# Medication Adherence for Haemophilia Patients: Outcome of Prophylaxis Treatment Intervention

**DOI:** 10.3390/healthcare9121702

**Published:** 2021-12-08

**Authors:** Fadzlin Mohd Mokhtar, Jameela Sathar, Hasniza Zaman Huri

**Affiliations:** 1 Department of Clinical Pharmacy and Pharmacy Practice, Faculty of Pharmacy, University Malaya, Kuala Lumpur 50603, Malaysia; fadzlin1617@gmail.com; 2 Department of Hematology, Ampang Hospital, Ministry of Health, Selangor 68000, Malaysia; jsathar@hotmail.com

**Keywords:** compliance, severe haemophilia A and B, Haemophilia Treatment Centre (HTC), Veritas-Pro

## Abstract

There have been various Haemophilia Treatment Centres (HTCs) set up worldwide with innovative blood factor stewardship programs. Pharmacists have been an extended part of stewardship programs providing daily rounds with haematologists, treatment plan modifications, and dosage adjustment recommendations. The Haemophilia Treatment Centres in Malaysia contain the Haemophilia Medication Therapy Adherence Clinic (HMTAC), which recruits adolescent and adult populations. There have not been any adherence studies conducted on pharmacist-steered HMTAC since initiation. The current research generates baseline data to produce treatment plans and intervention measures needed for therapy optimisation in the Malaysian population. This study also explores the relationship between medication adherence, bleeding rate, and comorbidity. This cross-sectional study involved retrospective and prospective data collection using the Validated Haemophilia Regimen Treatment Adherence Scale–Prophylaxis (VERITAS-Pro) in Ampang Hospital. The retrospective data collection included reviewing patients’ medical records, bleeding diaries, and VERITAS-Pro questionnaires pre-enrolment to HMTAC. Meanwhile, the prospective data collection was the VERITAS-Pro questionnaire administration post a minimum of three months after enrolment. The inclusion criteria were patients with severe haemophilia A and B with ages ≥18 years with self-administered prophylactic regimens for a minimum period of three months. There were six (5.8%) nonadherent participants, and 97 (94.2%) adhered to the preventive treatment. The subscale dosing and remembering and the total score of the VERITAS-Pro post-HMTAC showed a significant association with ABR. There was a significant mean reduction in the post-HMTAC compared to the pre-HMTAC score for the total score and subscales timing, remembering, skipping, and communicating. There was a significant association between the post-HMTAC adherence status and ABR. It can be concluded that the HMTAC service pioneered by the pharmacists in the National Referral Centre of Haematology is efficient in significantly improving the VERITAS-Pro scoring and then translating it into a high medication adherence rate. This study also highlights a significant correlation between post-HMTAC scores on their adherence with ABR and comorbidities.

## 1. Introduction

Haemophilia A (factor VIII deficiency) and Haemophilia B (factor IX deficiency) are hereditary bleeding conditions caused by clotting factor deficiencies. An x-linked recessive disorder primarily affects men, although females have the affected gene [1].

Haemophilia Treatment Centres (HTC) is a common name used worldwide for haemophilia clinics staffed by multidisciplinary healthcare professionals such as haematologists, orthopaedists, nurses, pharmacists, physical therapists, etc. However, there are variations in the staffing of HTCs. In the United States, HTCs operate under the Centre for Disease Control (CDC), centrally funded and available throughout the country. The HTCs, specialised centres, accommodate up to 70% of haemophilia patients in the country [2]. Various studies have shown that participation in HTCs reduces mortality and morbidity according to the Haemophsurvey A survey conducted by the CDC, which comprised 3000 haemophilia patients and showed that HTC participants were 40% less likely to die of a haemophilia-related complication than those who received care at a nonspecialised centre [2]. Similarly, participants of HTCs are 40% less likely to be hospitalised for any significant bleeding complications [3]. In Malaysia, Ampang Hospital is the designated National Referral Centre of Haematology and has operated a Haemophilia Medication Therapy Adherence Clinic (HMTAC) pioneered by pharmacists since 2007. Before establishing the HMTAC, prophylactic treatment was carried out in the haematology outpatient clinic. During outpatient treatment, the patient’s adherence and low annual bleeding rate were challenging to monitor due to the workforce and tools, such as a bleeding diary, pharmacy log, and infusion log. The HMTAC is conducted by a trained pharmacist who has been certified by the Ministry of Health Malaysia and has met the competency requirements as highlighted in the training program. The HMTAC guidelines require monthly follow-ups for six visits for optimum benefits to achieve the final goal of adherence. The main objective of the HMTAC is to educate the patients on their disease and how to optimally manage infusions of treatment to prevent long-term life and limb-threatening complications. Revision is done at every visit to ensure patients retain essential information regarding their treatment. Patients are also provided with a haemophilia kit consisting of an HMTAC diary, haemophilia card, cold box, ice packs, needles, syringes, alcohol swabs, gloves, and sharps bin upon enrolment [4]. 

In haemophilia, prophylaxis can be defined as administering clotting factors concentrated in anticipation of or to prevent bleeding [5]. This was first established in Sweden in 1958 and the Netherlands in 1968 [6,7]. For over six decades, the demonstrated benefits of prophylactic therapy have been reported in many studies, from preventing bleeding episodes to ultimately improving the quality of life in individuals with severe haemophilia [8,9,10]. Prophylactic treatment by routine factor VIII and factor IX infusions in patients with haemophilia was introduced to convert the bleeding phenotype from severe to moderate. Weekly infusions of preventive therapy might be painful to administer, especially if the vein is difficult to access. It is also commonly painful at the injection site and may lead to nonadherence in treatment. The existence of HMTAC helps to tackle nonadherence to the regimen of prophylactic treatment therapy, as it may lead to insufficient clotting factors and the development of haemophilia-related complications such as bleeding and chronic joint disease. 

The annualised bleeding rate (ABR) is an important variable to measure the effectiveness of prophylactic therapy. More recently, studies on the efficacy of preventive treatment in adolescents and adults have been published [11,12,13]. The prophylactic factor regimens have demonstrated clinical advantages in preventing and reducing bleeding episodes [13,14]. 

The adolescent and adult populations are recruited into the HMTAC service in Malaysia. There have not been any adherence studies conducted on pharmacist-steered HMTAC since its initiation. Using VERITAS-Pro, an assessment evaluating pre- and post-adherence, could elucidate adherence concerning time, dosage, planning, recall, skip, and communication. Therefore, this study would generate baseline data to produce treatment plans and intervention measures needed for therapy optimisation in this population. This study also aims to understand the relationship between medication adherence, bleeding, and comorbidity.

## 2. Materials and Methods

### 2.1. Study Sampling

The patients included in this study originated from active patients of the National Hematology Referral Centre in Ampang Hospital, Malaysia. The study is of a cross-sectional design that aims to establish the impact of pharmacist-run Haemophilia Medication Therapy Clinics on adherence to prophylactic treatment. The study was conducted following the principles of the Declaration of Helsinki. Ethical approval was granted by the Medical Research and Ethics Committee Ministry of Health (NMRR-18-3980-42265). Consent forms were received from participants before the initiation of the research.

For this study, retrospective baseline data were collected from patients aged 18 years and above who were newly referred to the National Haematology Referral Centre in Ampang Hospital between January 2018 and January 2020 with a diagnosis of severe haemophilia A and B in need of prophylactic treatment. Mild haemophilia is defined as factor activity between 5 and 40% of the normal regular factor activity; moderate haemophilia includes 1–5% of regular factor activity. Severe haemophilia is defined as factor activity of less than 1% in the bloodstream. A definitive diagnosis is made by measuring the FVIII or FIX activity and ruling out other pathological conditions or diseases with decreased FVIII or FIX activity. According to the factor activity level, severity is classified as severe, moderate, and mild [15]. All patients with a minimum 3-month enrolment in the HMTAC were administered VERITAS-Pro, a self-administered questionnaire, upon admission into the HMTAC (pre) and in January 2020 (post). 

### 2.2. Study Tool

The VERITAS-Pro, a self-administered validated questionnaire available in more than 30 languages, was used [16]. Questionnaires in English and Malay languages were used in this study based on the language proficiency of the respondents. The questionnaires comprise six subscales: time, dosage, prepare, recall, skip, and communicate. Each subscale consists of four items, with a total of 24 questions to be completed. 

Time refers to taking the treatment as scheduled, dose refers to infusing the prescribed dose, plan refers to the organisation around the supplies, remember refers to the missed infusions, skip deals with the quantities purposely not administered, communicate is for calling the treatment centre when needed, and treat refers to helping infusions when symptoms of bleeding occur. The total scores range from 24 to 120, and the subscale scores range from 4 to 20, with lower scores indicating higher adherence. Both questionnaires have shown good psychometric properties in US samples [17]. Scores on the VERITAS-Pro have correlated moderately to strongly with global adherence rating by primary infusers and medical staff [17]. Scores on the VERITAS-Pro were correlated with the percentage of recommended infusions administered from a web-based self-reporting log system and validated against pharmacy dispensation [17]. 

The total scores range from 24 to 120 and subscale scores range from 4 to 20, with lower scores indicating higher adherence [17]. Nonadherence is defined as a total cut-off score of 57, as determined in a previous study [17]. The total score is derived from the sum of all the subscales. Scores on VERITAS-Pro were correlated and validated with the pharmacy logs and recommended infusion logs [18]. The questionnaire was administered once during admittance into the HMTAC program and in 2020. Permission to use the questionnaire was obtained from the corresponding author before the start of the study.

Participants were dichotomised according to their adherence level using the cut-off score indicated in the original validation studies [16,19]. Patients with mild or moderate haemophilia A and B and an inhibitor were excluded from the study. Patients with inhibitors were excluded to prevent the clotting function of factors VIII and IX. Clinical and demographic data, such as age, gender, ethnicity, disease severity, comorbidity, medication, and the number of bleeding episodes, were manually collected via medical records from patients eligible for the VERITAS-Pro questionnaire. The data in patients’ diaries, including dosing, bleeding episodes, site of bleeding, and others, were documented.

### 2.3. Data Analysis

Data collected were analysed using the Statistical Package for Social Science software, version 26 (IBM, Armonk, NY, USA). Descriptive statistics, such as the mean, median, and standard deviation (SD), described continuous variables if normally distributed. Frequency and proportion were used to describe the categorical variables. The paired sample *t*-test was used to compare adherence scoring pre- and post-HMTAC. Multiple logistic regression analyses were performed to assess the association between post-subscale scoring and ABR following the adjustments for potential confounding factors such as participant characteristics and clinical relevance. A *p*-value of less than 0.05 was considered significant for the following analysis. 

## 3. Results

### 3.1. Study Population

**Figure 1:**
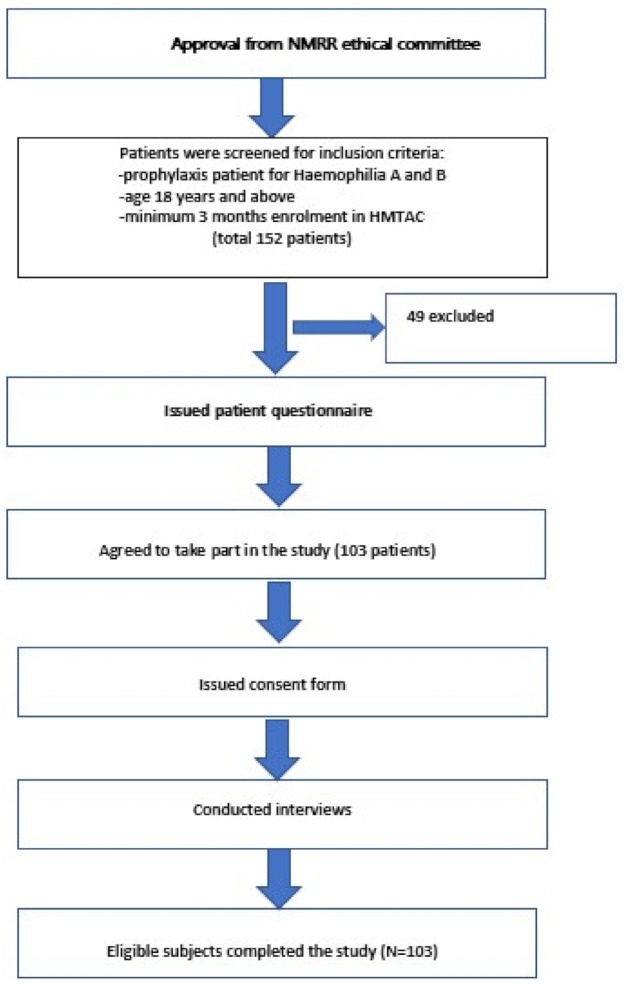
Flowchart of patient dispositions. Abbreviations: NMRR, National Medical Research Registry; HMTAC, Haemophilia Medication Therapy Adherence Clinic.

From January 2018 to January 2020, 152 patients were screened. Of these 152 patients, 49 respondents were excluded, as they did not fulfil the inclusion criteria. As a result, only 103 patients were eligible and completed the study. The distribution of patients is shown in Figure 1.

### 3.2. Demographic and Clinical Characteristic

A total of 103 male patients (100%) with severe haemophilia A and B enrolled in HMTAC completed the post-VERITAS-Pro questionnaire. The respondents’ ages were normally distributed with a mean ± SD of 33 ± 11.91 years, with a minimum age of 18 years and maximum age of 68 years. Patients’ ethnicities consisted of predominantly Malays (60) (58.3%), followed by Chinese (37) (35.9%), then Indian (5) (4.9%) and others (1) (1%). The mean VERITAS-Pro (*n* = 103) total score was 30 ± 20.04 (range, 26–68). The mean subscale score ranged from 4 (‘dose’) to 8 (‘communicate’).

The median for ABR was three, with a minimum of no bleeding per year and a maximum of 19 bleedings per year. The majority (*n* = 75) of the patients were diagnosed with comorbidities. The results also showed patients were diagnosed from zero comorbidity to a maximum of four comorbidities. Most of the participants had hepatitis C infection (*n* = 45), and other types of diseases have also been described, as shown in Table 1.

There were three types of dosing for prophylactic treatment involved in this study. Most of the participants (*n* = 40) were prescribed a twice-weekly regimen, followed by three times a week (*n* = 37) for severe haemophilia A. Only 26 participants were prescribed with a once-weekly dosing regimen for severe haemophilia B. In our study, there was no significant difference between the mean frequency per week of the adherent and nonadherent patients (*p* = 0.900).

Ethnicity, the presence of comorbidities, and the number of comorbidities were significantly associated with the adherence level. Chinese patients (*n* = 37) significantly adhered to prophylactic therapy (*p* = 0.013) compared with non-Chinese patients (*n* = 66), which consisted of Malay, Indian, and other ethnicities.

Patients with the presence of comorbidities (*p* = 0.043) and with more numbers of diseases (*p* = 0.026) significantly adhered to the prophylactic treatment (Table 1). There was no significant association between other characteristics such as age, dosing types, and duration in HMTAC and ABR with the adherence level (Table 1).

### 3.3. Factors Associated with Adherence and the Annual Bleeding Rate

There was a significant difference in the mean ABR between the adherent (94.2%) and nonadherent participants (5.8%) post-HMTAC. The mean bleeding rate of the adherent patients was significantly lower than the nonadherent (*p* = 0.005) group. Meanwhile, there was no significant difference for the mean ABR between the presence (72.81%) and absence (27.19%) of musculoskeletal disease. In the Kruskal–Wallis test, the null hypothesis was retained, in that the distribution of the annual bleeding rate was the same across the categories of types of dosing. The frequencies of the types of dosing for once a week (25.2%), twice a week (38.83%), and three times a week (35.92%) also did not influence the annual bleeding rate (Table 2).

A comparison between the pre and post-HMTAC annual bleeding rate could not be carried out due to missing and incomplete documentation during enrolment. 

However, there has been an associating adherence and ABR. Therefore, the adherence to prophylactic treatment for pre- and post-HMTAC was explored using the VERITAS-Pro questionnaire [18]. A paired *t*-test was conducted on all the subscale scores, and the total score showed a significant mean score reduction across all the subscale components of VERITAS-Pro except dosing and planning (Table 3).

Table 4 summarises the results of the multivariate analysis to examine the association between the adherence level post-HMTAC and ABR using scale scoring following multiple adjustments for potential confounders and mediators. There was a significant relationship between ABR with the VERITAS-PRO subscales of dosing and remember and the total score. However, they were no longer significant upon adjustment for ethnicity and the number of comorbidities (Table 4).

## 4. Discussion

This study, conducted in a HMTAC, showed that 94.2% of the participants (*n* = 97) adhered to the prophylactic treatment, with a minimum of 3 months of enrolment. Meanwhile, only 5.8% of participants (*n* = 6) did not comply with the treatment based on the cut-off level of 57 in the total score of VERITAS-Pro. This study reported a higher adherence level than previous studies, ranging from 17% to 82%, which were conducted in settings where multidisciplined health professionals such as doctors, nurses, and pharmacists were available [19,20,21]. The pharmacist-run HMTAC has proven to yield a high level of adherence post a minimum of three months of enrolment. According to the haemophilia protocol approved by the Ministry of Health, Malaysia, all enrolled patients were counselled by a pharmacist. Through the HMTAC service, pharmacists play a crucial role in imparting medication compliance and knowledge, recommending therapy regimens, and intervening in medication-related problems. There is evidence that knowledge contributes to and influences the medication adherence level of the patient. Several studies have found a positive association between knowledge and adherence [22,23,24,25]. 

The pre- and post-comparisons of the VERITAS-Pro results of the recruits in HMTAC enrolment showed a significant reduction in the mean VERITAS-Pro scores for the subscales timing, remembering, skipping, and communicating. The individual counselling with compliance and knowledge assessment during the initial visit could have motivated patients to acquire better achievements in these subscales. A lifestyle assessment was also conducted to better understand the patients for optimum counselling. A patient assessment checklist was reviewed on all visits, and previous counselling points were revised with the patient. Apart from counselling and knowledge assessments, the pharmacist also conducted checks of the patients’ haemophilia kits and ensured all items were adequate for better compliance [4]. Meanwhile, the dosing and planning comparisons of the pre- and post-scores showed nonsignificant differences due to the fixed nature of the prophylactic haemophilia medication dosing and differences in individual daily routines [26].

From this study, the median for ABR in HMTAC was three episodes. Typically, patients on prophylaxis had a mean ABR of 3.27 [27]. Furthermore, the adherence level showed a significant association with ABR in the VERITAS-Pro total score (*p* = 0.042). This result was the same as a previous study that suggested that a substantial number of bleeding events among adult patients could be eliminated through increased adherence [23]. The ‘dosing’ and ‘remembering’ subscales showed significant associations between adherence with ABR, with *p*-values of 0.025 and 0.018, respectively. These results may be due to the presence of a haemophilia diary, which was supplied upon recruitment of all patients into the HMTAC. The diaries consisted of relevant information regarding treatment and management, particularly of patients’ conditions, including medication dosing. Patients were more likely to remember taking the medication as stated in the diary, increasing their awareness and elevating their adherence levels.

In the subscales ‘timing’, ‘planning’, ‘skipping’, and ‘communication’, the bleeding frequencies were shown to have no association with the adherence levels. This may be due to the flexibility of patients’ individually tailored treatments. Patients were encouraged to tailor their treatments according to their lifestyles based on several principles that were pre-agreed on with their haematologists. Depending on their daily routine, patients would make their own decisions about changes in their injections without contacting the haemophilia centre. For example, a patient may infuse the correct dose on the recommended days of the week but infuse in the evening rather than the morning, as per the recommendations given, due to sports activity. This patient would be considered nonadherent to the above subscales but would not be considered nonadherent by clinicians.

Multivariate regression statistics were used to analyse the influence of the scoring subscales post-HMTAC (adherence) on the ABR. The adherence level was significantly greater in Chinese than non-Chinese patients, with *p* = 0.013 (Table 1). Patients of Chinese ethnicity were more adherent than those of non-Chinese ethnicities, which consisted of Malay, Indian, and others. Chinese patients are believed to be more health-conscious and adherent to health recommendations and medications as prescribed compared to other ethnic groups [28].

The presence and number of comorbidities were also found to be significantly associated with the adherence level, with *p*-values of 0.043 and 0.026, respectively (Table 1). The findings were consistent with those reported in a previous study, in which patients with comorbidities were more adherent to medications [29]. This may be related to age, as adult patients were more attentive to their treatment regimens because they were more aware of their mortality than younger patients; they have better knowledge of their illness and consequences if left untreated [30].

## 5. Conclusions

It can be concluded that the HMTAC service pioneered by the pharmacist in the National Referral Centre of Hematology is efficient in significantly improving VERITAS-Pro scoring, which translates into a high medication adherence rate. This study also highlights the significant correlation between post-HMTAC run by pharmacist adherence levels with ABR and comorbidities. 

## Figures and Tables

**Table 1:** Demographic and clinical characteristics of severe haemophilia A and B patients (*n* = 103).

Variable	Total Patients, *N* = 103 (%)	Adherent Patients, (%)	Non-Adherent Patients, (%)	*p*-Values
Age, year				0.144 ^a^
Mean ± SD	33.13 ± 11.91			
Min, Max	18.0; 68.0			
Ethnicity				**0.013 ^b^**
Malay	60 (58.3)	55 (56.7)	5 (83.3)	
Chinese	37 (35.9)	37 (38.1)	0 (0.0)	
Indian	5 (4.9)	4 (4.1)	1 (16.7)	
Others	1 (1.0)	1 (1.0)	0 (0.0)	
Presence of comorbidities				**0.043 ^b^**
Yes	58 (56.30)	56 (58.3)	1 (14.3)	
No	45 (43.68)	40 (41.7)	6 (85.7)	
Number of comorbidities				**0.026 ^a^**
Median ± SD	1 ± 0.94			
Min; Max	0; 4			
**Duration in HMTAC (months)**				0.339 ^a^
**Median ± SD**		9.00 ± 3.93	9.00 ± 4.07	
**Min, Max**		5, 36	5, 23	
Presence of Musculoskeletal disease				0.302 ^b^
Yes	32 (31.00)	29 (29.9)	3 (50)	
No	71 (68.93)	68 (70.1)	3(50)	
Types of comorbidities				
Hepatitis C	45 (43.69)			
Arthropathic	11 (10.68)			
Hypertension	6 (5.83)			
Diabetes Mellitus	5 (4.85)			
Retrovirus disease	2 (1.94)			
Fatty liver disease	2 (1.94)			
Epilepsy	2 (1.94)			
Psoriasis	2 (1.94)			
No comorbidities	28 (27.19)			
Annualised bleeding rates (ABR)				**0.029 ^a^**
Median ± SD	3 ± 4.12			
Min; Max	0; 19			
Types of Dosing (Post)				0.900 ^b^
Once a week *	26 (25.20)	24 (23.30)	2 (1.94)	
Twice a week **	40 (38.83)	37 (35.90)	3 (2.91)	
Three times a week **	37 (35.92)	35 (34.00)	2 (1.94)	

Values are expressed as the number (%) for categorical data; mean ± standard deviation. ^a^ Computed by one-way ANOVA; ^b^ computed by Pearson’s chi-square; bolded font indicates statistical significance at *p*-value < 0.05. * Haemophilia B; ** Haemophilia A.

**Table 2:** Factors associated with the annual bleeding rate.

	Annual Bleeding Rate (Mean ± SD)	*p*-Value
Adherence Post-HMTAC		0.005
Adherent	3.91 ± 3.989	
Nonadherent	7.67 ± 7.367	
Musculoskeletal disease		0.948
Yes	4.25 ± 4.558	
No	4.07 ± 4.015	
Types of Dosing		0.179
Once a week	5.08 ± 4.741	
Twice a week	3.13 ± 2.937	
Three times a week	4.54 ± 4.729	

**Table 3:** Subscale and total score of VERITAS-Pro pre- and post-HMTAC.

VERITAS-Pro Scale	Mean	Std. Deviation	*p*-Value
Timing score pre	6.66	2.936	0.003
Timing score post	5.52	1.846	
Dosing score pre	7.47	3.497	0.114
Dosing score post	5.10	1.866	
Planning score pre	7.26	2.364	0.064
Planning score post	6.29	2.243	
Remembering score pre	8.58	3.756	0.001
Remembering score post	7.00	2.853	
Skipping score pre	8.52	4.728	0.001
Skipping score post	5.91	2.716	
Communicating score pre	9.51	4.051	0.019
Communicating score post	8.20	3.510	
Total score pre	48.01	13.684	0.001
Total score post	38.03	9.848	

**Table 4:** Multivariate analysis on the influence of scoring of the subscales on annual bleeding rate post-HMTAC.

Adherence	Annual Bleeding Rate (ABR), Mean Difference (95% CI)		
	Unadjusted	Adjustment 1	Adjustment 2
Timing	0.177 (0.073)	0.179 (0.197)	0.189 (0.307)
Dosing	0.220 * (0.025)	0.222 (0.080)	0.222 (0.170)
Planning	0.058 (0.561)	0.058 (0.845)	0.074 (0.908)
Remembering	0.232 *(0.018)	0.233 (0.062)	0.236 (0.127)
Skipping	0.119 (0.232)	0.119 (0.490)	0.125 (0.664)
Communicating	0.036 (0.715)	0.037 (0.935)	0.064 (0.938)
Total score	0.201 * (0.042)	0.202 (0.124)	0.203 (0.241)

* Indicates significance at *p* < 0.05 (Adjustment 1: Ethnicity, Adjustment 2: Adjustment 1 + number of comorbidities).

## Data Availability

The study did not report any data.
